# The 5-HTTLPR Confers Susceptibility to Anorexia Nervosa in Han Chinese: Evidence from a Case-Control and Family-Based Study

**DOI:** 10.1371/journal.pone.0119378

**Published:** 2015-03-18

**Authors:** Jue Chen, Qing Kang, Wenhui Jiang, Juan Fan, Mingdao Zhang, Shunying Yu, Chen Zhang

**Affiliations:** Shanghai Mental Health Center, Shanghai Jiao Tong University School of Medicine, Shanghai, China; Sichuan University, CHINA

## Abstract

Accumulating evidence has implied that serotonin system dysfunction may be involved in the etiology of anorexia nervosa (AN). Serotonin-transporter-linked promoter region (5-HTTLPR) polymorphism is the genetic variant coding for the serotonin transporter and has a modulatory effect on its expression. This study aimed to investigate the possible association between the 5-HTTLPR and the susceptibility and severity of AN in Han Chinese using a case-control (255 patients and 351 controls) and family based study (198 trios). Eating disorder examination was used to measure the severity of AN behavioral symptoms. For the case-control study, the 5-HTTLPR showed significant association with AN in our sample (genotypic *P* = 0.03). The frequency of S allele was significantly higher in patients than that in controls (OR = 1.38, 95%CI: 1.06–1.79, *P* = 0.017). For the family-based study, the S allele of 5-HTTLPR was preferentially transmitted rather than non-transmitted from the parents to affected offspring (P = 0.013). The results of ANCOVA test revealed no significant association between the 5-HTTLPR polymorphism and severity of AN. Our findings suggested that 5-HTTLPR is able to confer susceptibility to AN in Han Chinese.

## Introduction

Anorexia nervosa (AN) is an eating disorder, characterized by excessive food restriction, typically arising from a morbid fear of weight gain that motivates patients to avoid eating. Although the pathogenesis of AN remains unknown, several lines of evidence supported the idea that AN is frequently associated with symptoms of anxiety, obsessive-compulsiveness and depression, and same pathophysiological mechanisms may underlie these symptoms [1].

Serotonin (5-HT) is a major neurotransmitter in the mammalian central nervous system (CNS). 5-HT modulates various CNS physiological activities including food intake, the sleep-wake cycle, cognition, and a wide repertoire of emotional behaviors [2]. Current studies have indicated that 5-HT system is involved in various psychiatric disorders and in the regulation of the feeling of satiety [3]. Consequently, it has been implied that 5-HT activity may be important in the physiopathology of AN [4].

Genetic epidemiological studies have assembled convincing evidence that AN is substantially influenced by genetic factors, and that the genetic component contributing to 50%∼70% of the variance in AN [5]. Therefore, several studies have attempted to search for genes in the 5-HT system that may be involved in the susceptibility to AN. One of the candidate genes is the serotonin transporter (5-HTT) gene, which encodes the human 5-HTT protein. A functional polymorphism in its 5’ regulatory promoter region (termed 5-HTTLPR: the 5-HTT gene-linked polymorphic region), consisting of two common alleles that correspond to a 44-base pair insertion (L allele) or deletion (S allele), regulates transcription of the 5-HTT gene [6]. The S allele of 5-HTTLPR polymorphism was found to reduce transcription efficiency for the 5-HTT gene, resulting in decreased 5-HTT expression and associated with a number of serotonin-related psychiatric disorders, such as bipolar disorder, depression, violent suicide.

So far, case-control studies have examined the association between 5-HTTLPR polymorphism and AN. Some preliminary findings have indicated a significant association of the S allele with AN [7,8], but such positive results were not replicated in other studies [9,10,11,12,13]. This fact may be due to population stratification, a limitation inherent to the population case-control study design. Therefore, the use of nuclear families is a critical feature being against population stratification [14]. In this study, we first carried out a two-phase study including both case-control and family-based analyses to examine the association between 5-HTTLPR polymorphism and AN in Han Chinese. In addition, it is well-known that AN is a complex and polygenic disorder, and specific genetic loci may contribute to the pathophysiology and phenotypic variability seen in AN [15]. Previous studies have indicated an influence of 5-HTTLPR on the severity of symptoms in psychiatric disorders, such as major depressive disorder [16], panic disorder [17], obsessive compulsive disorder [18], attention-deficit/hyperactivity disorder [19] and autism [20]. To the best of our knowledge, there is no report about the role of 5-HTTLPR in the severity of symptoms in AN. Therefore, the second aim of this study was to examine whether 5-HTTLPR was associated with specific symptom clusters in a sample of Han Chinese patients with AN.

## Methods

### Ethics statement

All participants provided written informed consent prior to inclusion in this project, and were treated in accordance with the Declaration of Helsinki. The study protocol and process was assessed and approved by the ethics committee at the Shanghai Mental Health Center. The Ethics Committee of the Shanghai Mental Health Center provided ethics approval for adolescent participants to provide their own consent rather than requiring consent for next of kin, caretakers or guardians.

### Subjects

We performed both case-control and family-based analyses in this study. For the family-based study, 198 trios consisting of 594 subjects were recruited. All probands from the family-based study were also part of the case-control study, which is detailed as follows. Their parents self-reported no history of AN. For the case-control study, a total of 255 patients with AN were recruited from the Department of Clinical Psychology, Shanghai Mental Health Center, Shanghai Jiao Tong University School of Medicine. Each patient was assessed and diagnosed by at least two senior psychiatrists according to DSM- IV (American Psychiatry Association). The sample consisted of 13 male and 242 female patients with a mean age at entry into the study of 19.4±4.7 years.

An additional 351 healthy control subjects (12 males and 339 females, mean age: 20.0±1.5 years) were recruited from hospital staff and students of School of Medicine in Shanghai, and then interviewed by a research psychiatrist according to the SCID-P [21]. Control subjects who reported a history of any psychiatric disorder or a metabolic, endocrine or gastrointestinal illness that could affect body weight were excluded from this study. All of the participants for each portion of the study were of Han Chinese origin.

### Clinical Measures

All measures were administrated at prior to enrollment. (1) Physical assessment: to calculate body mass index (BMI = kg/m^2^), participants’ weight and height were evaluated by a trained research assistant using a stadiometer. All patients were weighted in light indoor clothing, without shoes. (2) Eating disorder examination: eating attitudes and behaviors were specifically investigated by means of the Eating Disorder Examination Questionnaire (EDE-Q). The EDE-Q is a semi-structured investigator-based interview specifically devoted to the assessment of ED psychopathology, providing different levels of descriptive data concerning current ED psychopathology. The EDE-Q global scale was used to assess the severity of ED behavioral symptoms. The EDE-Q has previously demonstrated good reliability and validity [22,23].

### Genotyping

Blood samples were obtained from the participants and then Genomic DNA was isolated from whole blood using a Tiangen DNA isolation kit (Tiangen Biotech, Beijing, China). The analysis of the 5-HTTLPR variant was done using PCR as the method described by Xu et al [24]. The following primer pair was designed to span the variant region in the *5-HTT* promoter: 5′-GGCGTTGCCGCTCTGAATGC-3′ (forward) and 5′-GAGGGACTGAGCTGGACAACCAC-3′ (reverse). PCR was performed with the annealing temperature at 62°C for 33 cycles. The PCR products were subjected to electrophoresis in 2% agarose gels, stained with ethidium bromide and visualized under UV light.

### Statistical analysis

We used SPSS 17.0 (SPSS Inc., Chicago, IL, USA) to perform either the t-test or the chi-square test to compare demographic characteristics between the AN and control groups. For the case-control analysis, the Hardy-Weinberg equilibrium, allele and genotype frequencies of 5-HTTLPR polymorphism were determined using SHEsis (http://analysis.bio-x.cn) [25]. The family-based association test was performed on transmission disequilibrium test (TDT) program of Haploview 4.1. The possible effect of the 5-HTTLPR genotype on symptom severity was performed with ANCOVA by comparing the mean EDE-Q scores of each genotype. Variables that affected EDE-Q scores (i.e., sex, age, BMI, education and age at onset) were included as covariates. The statistical power of our sample size was calculated on Quanto program (Version 1.2.3, available at http://hydra.usc.edu/GxE). All tests were two-tailed and a significance level was set at *P*<0.05.

## Results

### Demographic and clinical features

Social-demographic and clinical characteristics of our sample are reported in Table 1. There were no significant differences between the patients with AN and control subjects with respect to age and sex. The patients with AN have significantly lower BMI than control subjects (16.2±2.7 vs 20.6±2.6, *P*≺0.01).

**Table 1:** Demographic features in AN patients and controls.

	Patients (n = 255)	Controls (n = 351)	χ^2^/t	*P*
Gender (M/F)	13/242	12/339	1.05	0.31
Age (years)	19.4±4.7	20.0±1.5	−1.43	0.15
BMI (kg/m2)	16.2±2.7	20.6±2.6	−14.35	<0.01
Education (years)	12.1±2.8	NA		
Age at onset (years)	16.7±3.5	NA		
	Patients (n = 177)			
EDE-Q total score	2.3±1.3	NA		
EDE-Q restraint	1.8±1.6	NA		
EDE-Q eating concern	2.5±1.5	NA		
EDE-Q weight concern	2.3±1.5	NA		
EDE-Q shape concern	2.8±1.6	NA		

BMI, body mass index; EDE-Q, Eating Disorder Examination Questionnaire.

### 5-HTTLPR and susceptibility to AN

5-HTTLPR has two common forms that are distinguished by a long and short allele depending on the insertion or deletion of 44bp. For the case-control study, the genotype distribution of the 5-HTTLPR polymorphism in control group was in accordance with Hardy-Weinberg equilibrium (*P* = 0.38). Comparisons of genotype and allele frequencies for the polymorphism between patients with AN and control subjects are presented in Table 2. The 5-HTTLPR showed significant association with AN in our sample (genotypic *P* = 0.03). The frequency of S allele was significantly higher in patients than that in controls (OR = 1.38, 95%CI: 1.06–1.79, *P* = 0.017). Family-based test showed that genotype distribution of 5-HTTLPR conformed to Hardy-Weinberg equilibrium in the parents and no Mendelian inheritance error was found. Allele frequencies analysis is shown in Table 3. The S allele of 5-HTTLPR was preferentially transmitted rather non-transmitted from the parents to affective offspring (*P* = 0.013). Under the assumption of a modest effect size and log additive model, the statistical power of our sample was more than 90% and 76% for the case-control and family-based analyses, respectively.

**Table 2:** Distribution of genotypes and alleles for the 5-HTTLPR polymorphism in AN patients and controls.

		Genotype N (%)					Allele N (%)				
5-HTTLPR	N	S/S	S/L	L/L	χ^2^	*P*	S	L	χ^2^	*P*	OR (95%CI)
Patients	255	158 (62.0)	77 (30.2)	20 (7.8)	6.89	0.03	393 (77.1)	117 (22.9)	5.68	0.017	1.38 (1.06–1.79)
Controls	351	180 (51.3)	138 (39.3)	33 (9.4)			498 (70.9)	204 (29.1)			

**Table 3:** Results of TDT analysis for 5-HTTLPR polymorphism in AN families.

	Allele			
Trios (N = 198)	S	L	χ^2^	*P*
Transmitted	72	45	6.23	0.013
Non-transmitted	45	72		

### 5-HTTLPR and severity of AN

A total of 177 unmedicated patients examined with the EDE-Q to evaluate pathological symptoms of AN. Then the scores of five factors of the EDE-Q were compared based on the genotypes of 5-HTTLPR. The results of ANCOVA test revealed no significant association between the 5-HTTLPR polymorphism and severity of AN (Table 4).

**Table 4:** Distribution of EDE-Q scores in the AN patients with the three 5-HTTLPR genotypes.

	5-HTTLPR				
EDE-Q	L/L (n = 13)	L/S (n = 60)	S/S (n = 104)	*F*	*P*
Restraint	2.65±1.74	1.89±1.76	1.57±1.48	2.45	0.09
Eating	2.42±1.62	2.81±1.57	2.28±1.40	1.89	0.15
Shape	2.91±1.89	3.00±1.63	2.58±1.48	0.98	0.38
Weight	2.48±1.68	2.24±1.47	2.23±1.47	0.15	0.86
Global	2.61±1.58	2.48±1.39	2.17±1.28	0.93	0.40

The *P*-values were adjusted for sex, age, BMI, education and age at onset.

EDE-Q, Eating Disorder Examination Questionnaire.

## Discussion

In the present study, we found significant difference in allele and genotype frequencies of 5-HTTLPR between the patient and control groups. Since the false-positive results may emerge due to the effects of population stratification in case-control investigation design, family-based study was carried out as an effective approach to follow up the findings from the case-control investigation [26]. In the family samples, the S allele of 5-HTTLPR was observed to be over-transmitted from heterozygous parents to probands. This confirmed the association of 5-HTTLPR and AN. Recently, analysis of the pooled results from independent studies by meta-analytic methods provided significant evidence for the association of S allele of 5-HTTLPR polymorphism with AN [27]. Our findings provided supportive evidence for this association in Han Chinese for the first time.

It is known that AN is a syndrome with a multifactorial etiopathogenesis involving psychological and biological factors [28]. Epidemiological data has pointed out that stressful experiences have important consequences for the development of AN [29,30]. At the molecular level, a number of experimental studies supported the idea about the 5-HTTLPR stress-sensitivity hypothesis [31] that 5-HTTLPR and stress jointly convey stable changes in serotonin transporter (SERT) expression [32]. The S carriers tend to exhibit lower expression of 5-HTT coupled with reduced reuptake of 5-HT from the synapse, and this may result in stronger psychopathological reactions to stressful experiences than those with L allele [33]. Brain imaging studies provide the potential for understanding neurotransmitter function, such as 5-HTT, and structural changes in relevant to human behaviors. Bailer et al. [34] used positron emission tomography (PET) imaging with McN5652 to assess the 5-HTT activity and found that women covered from restricting-type AN had elevated binding potential of 5-HTT in the dorsal raphe and anteroventral striatum in comparison to women who recovered from bulimia-type AN. These results implied that AN has neurobiological characteristics due to persistent disturbance of 5-HTT function. On the other side, a recent magnetic resonance imaging study has showed that in young women (mean age = 25.6 years), the effect of stress on structural connectivity between the left hippocampus and both the amygdala and the putamen may be regulated by a 5-HTTLPR genotype-mediated mechanism of implicit learning after negative experiences [35]. Meanwhile, neuroimaging studies have shown that structural abnormalities in these brain regions are commonly seen in patients with AN [36,37,38]. Given the regulatory effects of 5-HTTLPR on life stress, the aforementioned results suggested that changes in brain structure among patients with AN may be influenced by stressful experiences and 5-HTTLPR is likely to play an important role in this process.

As a secondary aim, this study examined whether 5-HTTLPR was associated with specific symptom clusters. Our sample was divided into three groups on the basis of subject genotype and EDE-Q score differences between subjects with the different genotypes were analyzed. Data obtained showed that the 5-HTTLPR polymorphism is not associated with the severity of AN. This seems to suggest 5-HTTLPR is not likely to facilitate the possibility of developing these psychopathological traits. AN is one of the most heritable complex disorders. Previous studies have illustrated the influence of dopaminergic and serotonergic system on the psychopathological features displayed by patients with AN [11,39,40,41]. With regard to serotonin genes, Gervasini et al. [42] proposed that polymorphisms in serotonin gene system and especially their epistatic interactions leading to low neurotransmitter activity may be associated with a higher severity of psychopathological traits in eating disorder patients. Several lines of evidence suggested that interaction of 5-HTTLPR and serotonin genes exert important biological effects in multiple psychiatric disorders [43,44,45,46]. Therefore, further studies are needed that include wider arrays of serotonin genes to fully ascertain the influence of 5-HTTLPR on the psychopathological traits of AN.

When interpreting the results of this study, we would be remiss in not noting some limitations. Aside from the small sample size used in this study, there are two potential limitations that are worth noting. First, while subjects were all of Chinese origin, we could not completely exclude the possibility of a population structure effect in our sample [47]. Second, the occurrence of AN—being a polygenic disorder—is widely known to depend on the interaction of multiple factors [48]. Usually no single gene is responsible for this disorder, and the methods used in individual studies may have limited power to detect what may be a potentially small effect. Third, the case-control portion and the family-based one were not independent. All probands from the family-based were also part of the case-control study. Last, the control subjects were recruited from hospital staff and students of the School of Medicine in Shanghai, even who were psychiatrically screened for mental disorder, and the samples were not representative of the general population.

In conclusion, our results presented herein indicated that 5-HTTLPR is able to confer susceptibility to AN in Han Chinese. For validation, further independent studies in larger samples are needed to confirm these preliminary observations and to establish other possible implications, such as the psychopathological traits of this disorder.

## References

[1] Godart NT, Perdereau F, Rein Z, Berthoz S, Wallier J, Jeammet P, et al (2007) Comorbidity studies of eating disorders and mood disorders. Critical review of the literature. J Affect Disord 97: 37–49. 16926052 10.1016/j.jad.2006.06.023

[2] Hoyer D, Hannon JP, Martin GR (2002) Molecular, pharmacological and functional diversity of 5-HT receptors. Pharmacol Biochem Behav 71: 533–554. 11888546 10.1016/s0091-3057(01)00746-8

[3] Gauthier C, Hassler C, Mattar L, Launay JM, Callebert J, Steiger H, et al (2014) Symptoms of depression and anxiety in anorexia nervosa: Links with plasma tryptophan and serotonin metabolism. Psychoneuroendocrinology 39: 170–178. 10.1016/j.psyneuen.2013.09.009 24135616

[4] Kaye WH, Fudge JL, Paulus M (2009) New insights into symptoms and neurocircuit function of anorexia nervosa. Nat Rev Neurosci 10: 573–584. 10.1038/nrn2682 19603056 PMC13038070

[5] Klump KL, Gobrogge KL (2005) A review and primer of molecular genetic studies of anorexia nervosa. Int J Eat Disord 37 Suppl: S43–48. 15852319 10.1002/eat.20116

[6] Heils A, Teufel A, Petri S, Stober G, Riederer P, Bengel D, et al (1996) Allelic variation of human serotonin transporter gene expression. J Neurochem 66: 2621–2624. 8632190 10.1046/j.1471-4159.1996.66062621.x

[7] Matsushita S, Suzuki K, Murayama M, Nishiguchi N, Hishimoto A, Takeda A, et al (2004) Serotonin transporter regulatory region polymorphism is associated with anorexia nervosa. Am J Med Genet B Neuropsychiatr Genet 128B: 114–117. 15211642 10.1002/ajmg.b.30022

[8] Di Bella DD, Catalano M, Cavallini MC, Riboldi C, Bellodi L (2000) Serotonin transporter linked polymorphic region in anorexia nervosa and bulimia nervosa. Mol Psychiatry 5: 233–234. 10889521 10.1038/sj.mp.4000689

[9] Fumeron F, Betoulle D, Aubert R, Herbeth B, Siest G, Rigaud D. (2001) Association of a functional 5-HT transporter gene polymorphism with anorexia nervosa and food intake. Mol Psychiatry 6: 9–10. 11244478 10.1038/sj.mp.4000824

[10] Hinney A, Barth N, Ziegler A, vonPrittwitz S, Hamann A, Hennighausen K, et al (1997) Serotonin transporter gene-linked polymorphic region: Allele distributions in relationship to body weight and in anorexia nervosa. Life Sci 61: Pl295–Pl303. 9395256 10.1016/s0024-3205(97)00888-6

[11] Rybakowski F, Slopien A, Dmitrzak-Weglarz M, Czerski P, Rajewski A, Hauser J. (2006) The 5-HT2A-1438 A/G and 5-HTTLPR Polymorphisms and personality dimensions in adolescent anorexia nervosa: Association study. Neuropsychobiology 53: 33–39. 16397402 10.1159/000090701

[12] Sundaramurthy D, Pieri LF, Gape H, Markham AF, Campbell DA (2000) Analysis of the serotonin transporter gene linked polymorphism (5-HTTLPR) in anorexia nervosa. Am J Med Genet 96: 53–55. 10686552

[13] Lauzurica N, Hurtado A, Escarti A, Delgado M, Barrios V, Morandé G, et al (2003) Polymorphisms within the promoter and the intron 2 of the serotonin transporter gene in a population of bulimic patients. Neurosci Lett 352: 226–230. 14625025 10.1016/j.neulet.2003.08.058

[14] Aberg KA, Liu Y, Bukszar J, McClay JL, Khachane AN, Andreassen OA, et al (2013) A comprehensive family-based replication study of schizophrenia genes. JAMA Psychiatry 70: 573–581. 23894747 10.1001/jamapsychiatry.2013.288 PMC5297889

[15] Monteleone P, Maj M (2008) Genetic susceptibility to eating disorders: associated polymorphisms and pharmacogenetic suggestions. Pharmacogenomics 9: 1487–1520. 10.2217/14622416.9.10.1487 18855537

[16] Mehta D, Quast C, Fasching PA, Seifert A, Voigt F, Beckmann MW, et al (2012) The 5-HTTLPR polymorphism modulates the influence on environmental stressors on peripartum depression symptoms. J Affect Disord 136: 1192–1197. 10.1016/j.jad.2011.11.042 22209125

[17] Lonsdorf TB, Ruck C, Bergstrom J, Andersson G, Ohman A, Schalling M, et al (2009) The symptomatic profile of panic disorder is shaped by the 5-HTTLPR polymorphism. Prog Neuropsychopharmacol Biol Psychiatry 33: 1479–1483. 10.1016/j.pnpbp.2009.08.004 19683026

[18] Denys D, Van Nieuwerburgh F, Deforce D, Westenberg HGM (2006) Association between serotonergic candidate genes and specific phenotypes of obsessive compulsive disorder. J Affect Disord 91: 39–44. 16443280 10.1016/j.jad.2005.12.011

[19] Kotte A, Faraone SV, Biederman J (2013) Association of Genetic Risk Severity With ADHD Clinical Characteristics. Am J Med Genet B Neuropsychiatr Genet 162B: 718–733. 10.1002/ajmg.b.32171 24132904

[20] Gadow KD, DeVincent CJ, Siegal VI, Olvet DM, Kibria S, Kirsch SF, et al (2013) Allele-specific associations of 5-HTTLPR/rs25531 with ADHD and autism spectrum disorder. Prog Neuropsychopharmacol Biol Psychiatry 40: 292–297. 10.1016/j.pnpbp.2012.10.019 23123360 PMC3522768

[21] Zhang C, Zhang J, Fan J, Cheng W, Du Y, Yu S, et al (2014) Identification of ANKK1 rs1800497 variant in schizophrenia: New data and meta-analysis. Am J Med Genet B Neuropsychiatr Genet 165B: 564–571. 10.1002/ajmg.b.32259 25073965

[22] Grilo CM, Masheb RM, Lozano-Blanco C, Barry DT (2004) Reliability of the Eating Disorder Examination in patients with binge eating disorder. Int J Eat Disord 35: 80–85. 14705160 10.1002/eat.10238

[23] Rizvi SL, Peterson CB, Crow SJ, Agras WS (2000) Test-retest reliability of the eating disorder examination. Int J Eat Disord 28: 311–316. 10942917 10.1002/1098-108x(200011)28:3<311::aid-eat8>3.0.co;2-k

[24] Xu J, Cheng YQ, Chen B, Bai R, Li S, Xu XF, et al (2013) Depression in systemic lupus erythematosus patients is associated with link-polymorphism but not methylation status of the 5HTT promoter region. Lupus 22: 1001–1010. 10.1177/0961203313498793 23893825

[25] Shi YY, He L (2005) SHEsis, a powerful software platform for analyses of linkage disequilibrium, haplotype construction, and genetic association at polymorphism loci. Cell Res 15: 97–98. 15740637 10.1038/sj.cr.7290272

[26] Zhang C, Fang Y, Xie B, Cheng W, Du Y, Wang D, et al (2009) DNA methyltransferase 3B gene increases risk of early onset schizophrenia. Neurosci Lett 462: 308–311. 10.1016/j.neulet.2009.06.085 19576953

[27] Lee Y, Lin PY (2010) Association between Serotonin Transporter Gene Polymorphism and Eating Disorders: A Meta-Analytic Study. Int J Eat Disord 43: 498–504. 10.1002/eat.20732 19708070

[28] Castellini G, Ricca V, Lelli L, Bagnoli S, Lucenteforte E, Faravelli C, et al (2012) Association between serotonin transporter gene polymorphism and eating disorders outcome: A 6-year follow-up study. Am J Med Genet B Neuropsychiatr Genet 159B: 491–500. 10.1002/ajmg.b.32052 22488946

[29] Fairburn CG, Cooper Z, Doll HA, Welch SL (1999) Risk factors for anorexia nervosa—Three integrated case-control comparisons. Arch Gen Psychiatry 56: 468–476. 10232302 10.1001/archpsyc.56.5.468

[30] Pike KM, Hilbert A, Wilfley DE, Fairburn CG, Dohms FA, Walsh BT, et al (2008) Toward an understanding of risk factors for anorexia nervosa: a case-control study. Psychol Med 38: 1443–1453. 18070371 10.1017/S0033291707002310 PMC2669537

[31] Caspi A, Hariri AR, Holmes A, Uher R, Moffitt TE (2010) Genetic sensitivity to the environment: the case of the serotonin transporter gene and its implications for studying complex diseases and traits. Am J Psychiatry 167: 509–527. 10.1176/appi.ajp.2010.09101452 20231323 PMC2943341

[32] Wankerl M, Miller R, Kirschbaum C, Hennig J, Stalder T, Alexander N. (2014) Effects of genetic and early environmental risk factors for depression on serotonin transporter expression and methylation profiles. Transl Psychiatry 4: e402 10.1038/tp.2014.37 24937096 PMC4080318

[33] Li Q (2006) Cellular and molecular alterations in mice with deficient and reduced serotonin transporters. Mol Neurobiol 34: 51–65. 17003521 10.1385/mn:34:1:51

[34] Bailer UF, Frank GK, Henry SE, Price JC, Meltzer CC, Becker C, et al (2007) Serotonin transporter binding after recovery from eating disorders. Psychopharmacology (Berl) 195: 315–324. 17690869 10.1007/s00213-007-0896-7

[35] Favaro A, Manara R, Pievani M, Clementi M, Forzan M, Bruson A, et al (2014) Neural signatures of the interaction between the 5-HTTLPR genotype and stressful life events in healthy women. Psychiatry Res 223: 157–163. 10.1016/j.pscychresns.2014.05.006 24914006

[36] Fuglset TS, Endestad T, Landro NI, Ro O (2015) Brain structure alterations associated with weight changes in young females with anorexia nervosa: a case series. Neurocase 21: 169–177. 10.1080/13554794.2013.878728 24460514

[37] Mainz V, Schulte-Ruther M, Fink GR, Herpertz-Dahlmann B, Konrad K (2012) Structural Brain Abnormalities in Adolescent Anorexia Nervosa Before and After Weight Recovery and Associated Hormonal Changes. Psychosom Med 74: 574–582. 10.1097/PSY.0b013e31824ef10e 22511729

[38] Giordano GD, Renzetti P, Parodi RC, Foppiani L, Zandrino F, Giordano G, et al (2001) Volume measurement with magnetic resonance imaging of hippocampus-amygdala formation in patients with anorexia nervosa. J Endocrinol Invest 24: 510–514. 11508785 10.1007/BF03343884

[39] Ricca V, Nacmias B, Boldrini M, Cellini E, di Bernardo M, Ravaldi C, et al (2004) Psychopathological traits and 5-HT2A receptor promoter polymorphism (-1438 G/A) in patients suffering from Anorexia Nervosa and Bulimia Nervosa. Neurosci Lett 365: 92–96. 15245785 10.1016/j.neulet.2004.04.057

[40] Gervasini G, Gordillo I, Garcia-Herraiz A, Flores I, Jimenez M, Monge M, et al (2013) Influence of dopamine polymorphisms on the risk for anorexia nervosa and associated psychopathological features. J Clin Psychopharmacol 33: 551–555. 10.1097/JCP.0b013e3182970469 23775054

[41] Nisoli E, Brunani A, Borgomainerio E, Tonello C, Dioni L, Briscini L, et al (2007) D2 dopamine receptor (DRD2) gene Taq1A polymorphism and the eating-related psychological traits in eating disorders (anorexia nervosa and bulimia) and obesity. Eat Weight Disord 12: 91–96. 17615493 10.1007/BF03327583

[42] Gervasini G, Gordillo I, Garcia-Herraiz A, Flores I, Jimenez M, Monge M, et al (2012) Polymorphisms in serotonergic genes and psychopathological traits in eating disorders. J Clin Psychopharmacol 32: 426–428. 10.1097/JCP.0b013e3182539f2b 22561477

[43] Antypa N, Serretti A, Rujescu D (2013) Serotonergic genes and suicide: a systematic review. Eur Neuropsychopharmacol 23: 1125–1142. 10.1016/j.euroneuro.2013.03.013 23742855

[44] Lohoff FW, Narasimhan S, Rickels K (2013) Interaction between polymorphisms in serotonin transporter (SLC6A4) and serotonin receptor 2A (HTR2A) genes predict treatment response to venlafaxine XR in generalized anxiety disorder. Pharmacogenomics J 13: 464–469. 10.1038/tpj.2012.33 22907732

[45] Blaya C, Salurn GA, Moorjani P, Seganfredo AC, Heldt E, Leistner-Segal S, et al (2010) Panic disorder and serotonergic genes (SLC6A4, HTR1A and HTR2A): Association and interaction with childhood trauma and parenting. Neurosci Lett 485: 11–15. 10.1016/j.neulet.2010.08.042 20817074

[46] Saiz PA, Garcia-Portilla MP, Arango C, Morales B, Alvarez V, Coto E, et al (2007) Association study of serotonin 2A receptor (5-HT2A) and serotonin transporter (5-HTT) gene polymorphisms with schizophrenia. Prog Neuropsychopharmacol Biol Psychiatry 31: 741–745. 17291660 10.1016/j.pnpbp.2007.01.012

[47] Zhang C, Cai J, Zhang J, Li Z, Guo Z, Zhang X, et al (2014) Genetic modulation of working memory deficits by ankyrin 3 gene in schizophrenia. Prog Neuropsychopharmacol Biol Psychiatry 50: 110–115. 10.1016/j.pnpbp.2013.12.010 24361380

[48] Sullivan PF, Daly MJ, O'Donovan M (2012) DISEASE MECHANISMS Genetic architectures of psychiatric disorders: the emerging picture and its implications. Nat Rev Genet 13: 537–551. 10.1038/nrg3240 22777127 PMC4110909

